# Improving Care of People With Serious Medical Illness—An Economic Research Agenda for Palliative Care

**DOI:** 10.1001/jamahealthforum.2021.4464

**Published:** 2022-01-04

**Authors:** Mireille Jacobson, Peter May, R. Sean Morrison

**Affiliations:** Aging Program, University of Southern California Schaeffer Center for Health Policy & Economics, University of Southern California Leonard Davis School of Gerontology, Los Angeles; Centre for Health Policy and Management, Trinity College Dublin, the Irish Longitudinal Study on Ageing, Dublin, Ireland; Brookdale Department of Geriatrics and Palliative Medicine, Icahn School of Medicine at Mount Sinai, Patty and Jay Baker National Palliative Care Center, New York, New York

In 2019, the US spent nearly $4 trillion on health care, almost 20% of the overall economy and far more than other high-income nations.^1,2^ The top 10% of spenders, many with serious chronic illness, account for two-thirds of this cost.^3^ Despite such high spending, many patients with serious illnesses (and their families) experience unmanaged pain and other symptoms, unmet needs, and treatment that is inconsistent with their preferences.^4,5^ Nearly 30% of patients with serious illness report being sent to undergo duplicate tests or diagnostics, 22% feel that hospital staff are unresponsive to their needs, and more than 60% have felt anxious, confused, or helpless when receiving care.^6^ More people are living with serious illness as the population ages, and patients with socioeconomic disadvantages bear the greatest burdens of poor care.^7^

Policy makers and the US public are largely aligned on the need to improve care for patients with serious illness while curtailing wasteful spending and easing burdens on family caregivers. By 1 estimate, the value of unpaid US family caregiving for people with serious illness exceeds $300 billion annually.^8^ Studies to date suggest that palliative care could help increase the value, defined as some combination of better quality and lower costs, of US health care spending. Palliative care, which “attends to the physical, functional, psychological, practical, and spiritual consequences of a serious illness,” improves care quality.^9^ However, without robust economic evidence to calculate the value equation, including costs and benefits, the current paradigm of high costs, poor outcomes, and growing inequities will persist for people with serious illnesses.

## Research Priorities to Build the Economic Evidence Base in Palliative Care

In 2020, the USC Schaeffer Center for Health Policy & Economics (Los Angeles, California) convened an expert panel to develop a consensus-driven economic research agenda for palliative care. The panel, which included experts in health economics and palliative and geriatric medicine, as well as payers, policy makers, and research funders, noted that current evidence gaps have not occurred incidentally. Substantial challenges to research on palliative care include stakeholder information gaps and variation in delivery models. As a result, to our knowledge, identifying when patients would benefit from specific palliative care interventions, how to measure those benefits, and how benefits vary over time has not been well studied. Our hope is that the research agenda (https://healthpolicy.usc.edu/evidence-base/improving-care-of-people-with-serious-medical-illness-an-economic-research-agenda-for-palliative-care/), which includes questions on supply-side, demand-side, and methodological factors (Table), will spur researchers and funders to undertake studies designed to identify the costs and benefits of palliative care.

### Supply-side Factors: Payment Policy and Access

Reimbursement policy affects care delivery and patient treatment choices. A better understanding of current payment practices could help identify barriers to high-value palliative care. Medicare is the major US payer of palliative care services. Fee-for-service Medicare, which covers almost 3 in 5 beneficiaries, generally supports palliative care in inpatient settings but not in ambulatory settings, including patients’ homes. Some Medicare Advantage plans offer palliative care services, but the lack of uniform quality standards and accreditation requirements contributes to highly variable service delivery. Studies are needed to evaluate whether and how alternative payment models can incentivize palliative care delivery across settings.

### Demand-side Factors: Preferences, Insurance Benefits, and Future Needs

Economic analyses of palliative care demand are partially limited because of disagreement over how to measure what people value. The large role that insurance plays in access to palliative care, information gaps among patients and clinicians, and family financial incentives and out-of-pocket costs also make analyzing demand complex. New studies in outcome measurement and goal-concordant care should be a priority. Even defining palliative care need and associated costs is contested. For example, the current specialist palliative care workforce is insufficient to meet the needs of all patients with advanced cancer as recommended by the American Society of Clinical Oncology, let alone those with other serious illnesses. Econometric analysis of existing data could guide palliative care service development, delivery, and workforce needs.

### Methods and Data: Evaluation of Quality, Cost, and Outcomes

Most randomized clinical trials have found that palliative care interventions improve quality of life but generally have found no effect on costs.^9^ However, such trials were rarely sized to assess cost effects. Observational studies often estimate large cost savings but can experience selection bias. The application of quasiexperimental methods to routinely collected data may help in the short term. Longer term, bridging data gaps through improved linkage of existing data, collecting new data with good generalizability outside of small trials, and facilitating data sharing would add enormous value. Such research could identify where, when, and how palliative care, including unpaid care, is provided and elicit patient preferences for types of palliative care. Large pragmatic clinical trials of palliative care that are embedded within existing practice also can potentially illuminate patient outcomes and costs to patients, payers, and society.

## Conclusions

Evidence of poor outcomes, high costs, and persistent inequities for serious medical illness suggests substantial room for improvement. Our consensus-driven palliative care research agenda aims to encourage researchers and funders to build the economic evidence base needed by policy makers and public and private payers to improve care across settings and conditions.

## Figures and Tables

**Table.** Economic Research Agenda for Palliative Care: Examples of Questions

Factors	Questions	
Supply-side factors: clinician payment policy and access	What incentives can encourage adoption of promising palliative care models, and what research designs can track individual and system outcomes?	What are the potential effects of different payment approaches across settings and payers on utilization, spending, and quality of care for individuals with serious illness?
Demand-side factors: preferences, insurance benefits, and future needs	What actions are needed to achieve equitable access to care consistent with patient preferences across different groups (including racial and ethnic minority groups)?	As populations age and care becomes more complex, how should we define palliative care need in the population and estimate that need in the coming decades?
Methods and data: evaluation of quality, cost, and experience outcomes	What data exist to define relevant populations; identify receipt of relevant interventions; estimate costs to payers, families, and patients; and measure outcomes and experiences of families and patients? What are the barriers to accessing and linking these data in government and the private sector?	What are the opportunities to evaluate palliative care programs using current econometric methods? Recent innovations to model different doses and program rollouts, as well as other approaches, such as pragmatic randomized clinical trials, may improve researcher capacity to isolate the effect of service and policy changes.
